# A Case of Typhoid Fever Marked by Some Peculiarities

**Published:** 1853-05

**Authors:** William Kenney

**Affiliations:** Millersburgh, Ky.


### Art. V.—*A Case of Typhoid Fever marked by some peculiarities.*

By William Kenney, M. D., of Millersburgh, Ky.

The subject of this report, Reuben T. Batson, forty-one years of age, a stout and athletic man, a native of this place and a carpenter by trade, of previous good health, given to a moderate use of ardent spirits, after some two weeks of fatigue and loss of sleep in attention to an afflicted little son, was taken on Wednesday morning, April 13, with chilliness, pain in the head and back, fever, general muscular soreness, moderate thirst, a coated tongue and constipated bowels with a pulse moderately full and 96 to the minute. His urine free and high colored. The pain in the back precluding the possibility of sleep or quietude, was of a severe and lancinating character, situated over the junction of the last lumbar vertebra with the sacrum, and so very excrutiating that he kept up a constant wincing and twitching as if to moderate or throw off its violence.

In this condition we found him and directed rest in a horizontal position, a mercurial carthartic to be followed in six or eight hours by oil, free sponging of the surface, acidulated and cooling drinks with cold applications to the head, opiate embrocations and flannels wrung out of hot water to the seat of pain in the back.

Thursday, 14th. Patient slept none last night, as he had failed to do since Monday night—medicine acted very well, two operations, the last bilious and consistent, urinates more frequently and less freely. Pain of head much relieved, no fever, surface soft and of natural temperature, pulse 84, tongue pale and covered with a heavy light colored coat of mucus wearing the indentations of the teeth. No appetite, a little enfeebled but thinks he would be well if he could sleep and get relieved of the pain in the back, which continues more of a nervous character, aggravated, and described as a pleuritic stitch on prolonged inhalation.

To-day, in anticipation of a return of fever we prescribed sul. quinine and directed a blister to the seat of pain: the denuded surface, to be covered after vesication by lint saturated with tinct. opii, and left a compound powder of ipecac, calomel, hyd. cum cret, and sul. morphia for night, with the privilege of some fluid nourishment during the day.

Here the peculiar character of the pain in the back led to an inquiry as to the probablility of his having received an injury, in this region, at any time. His reply was that some years ago in lifting he received an injury that lamed him for a few days and that in the last few weeks he gave it another wrench that hurt him some but did not stop him from work, but upon manual examination of the spinal column no tenderness was developed, except of a slight character, over the seat of pain that gave rise to pain of a shooting character over the trochanter major and down the front of the thigh to the knee.

Friday, 15th. No fever on yesterday, and towards noon profuse perspiration, of a clammy character, covered the surface. Delirium presented and a sleepless night was passed, the patient complaining much of pain.

To-day perspiration continues, features blanched, skin corrugated, extremities cold, tongue clammy and protruded with a tremulous motion, pulse 112 to the minute, urine high colored with a lateritious deposite, bowels moved, operations rather thin, appears restless and anxious, calls for drinks and takes nourishment. Delirium continues and he casts the eyes exploringly over the room, imagining the presence of strangers who design him mischief, feels his bed moved by some one secreted beneath it, calls out to persons as if passing in the distance, thinks himself away and wants to go home, answers questions promptly and with precision, shrinks from contact with some unknown person whom he imagines in bed with him, and with his arms, tremulous and partially extended, rolls up the bed-clothing and speaks of it as a child, a saw, a piece of dressed timber or his afflicted limb; loquacious, the mind passing rapidly from one matter to another and yet not altogether inattentive to what is going on about him. Starts as if spoken to, pupils contracted, head cool and the pain in the back now removed and apparently of a most distressing character over the trochanter major of the right side, the limb immoveably extended and the very touch of the toes excruciatingly painful.

The case growing more urgent, omitted quinine owing to idiosyncrasy of patient, and directed camph. mixt, arom. sp. ammon, tinct. opii in brandy every few hours, with nourishing diet, and the pain to be followed by blisters and anodyne dressings. At night a drachm of tinct., opii, if no sleep before, and the room kept quiet.

Saturday, 16th. Restless during the night, no sleep, delirium continues and perspiration profuse, pulse frequent and feeble. The pain from trochanter with unmitigated severity now appears over the inguinal region, feels some uneasiness yet in the back, has taken nourishment, but objects to the brandy as producing excitement and a glow throughout the system, bowels moved twice, the operations thin, urine scant and frequently passed with pain.

Prescription continued, the amount of brandy increased and directed gr. ss s. morphia at night.

Sunday, 17th. No sleep, delirium and a free and fluid state of the bowels continue with increased feebleness, and an icterode appearance of the conjunctiva; tongue coated brown in the centre, ardent urine, restless, and applies the hand frequently to the head, pulse 124, and thready. A strong ammoniacal odor emanates from the body, and the patient says he is cold.

Camph. mixt., brandy, &c., continued with directions that he should have a stream of cold water played upon the head, and ij. grs. of s. morphia at night.

Monday, 18th. Slept the greater portion of last night, and expresses himself, this morning, as being much better, feels drowsy and calls for food and drink, bowles moved twice, the last operation approaching something of a more consistent character, tongue moist and still coated, pulse 96 and compressible, surface cool and clammy, urinates less frequently, and directs your attention to the pain of the thigh and leg, though of a much less violent character, and can permit of its being cautiously moved. He frequently speaks of the soreness of his back.

The patient has now, apparently, moments of rationality alternating with delirium of the more usual typhoid form, with restlessness and anxiety. The eye is dull in its expression, pupils contracted, and features of a dusky sallow cast, bowels slightly tender and tympanitic.

Directed brandy and nourishment through the day, warm fomentations to the abdomen, with gtt. X of tinct. opii occasionally, and hyd. cum cret. ipecac and S. Morphia at night.

Tuesday, 19th. Slept well last night, feels more cheerful and less stupor than yesterday, yet sleep is disturbed by muttering and delirium, throughout the day. He has a perfect recollection as he has continued to have of his friends who visit him, and not unfrequently asks what is going on down in town. The surface is cool, pulse 84, with a little more volume. No operation, urine tolerably free.

Prescription of yesterday continued with the addition of friction and sinapisms to the surface and extremities.

Wednesday, 20th. Our patient passed a miserable night with an aggravation of the more unfavorable symptoms over yesterday; has tremor of the extremities, a frequent and feeble pulse with the condition of the surface as heretofore. The left nates, the seat of pain, much swollen, and the superincumbent structure edematous and of a jaundiced hue. He refers to pain of the back, and exhibits more of the results of that morbid action going on in the system, with a state of mind that

> "Doth by the idle comments that it makes
> Foretell the ending of mortality."

The tongue coated brown with red edge and tip, bowels moved last night, the operations offensive, and continue of a fluid nature.

Directed brandy, camphor and S. Morphia, with some nourishment and anodyne enemata.

Thursday, 21st. No sleep last night, restless and delirious, skin cool and clammy, pulse 112, refuses food, respiration somewhat quickened, enlargement of the nates, with pain and tenderness much increased and fluctuation with a deposit of matter deep seated. No complaint now of left hip and yet unable to use it.

Continue brandy, camphor and morphia.

Friday, 22d. Patient moribund, pulse 124, respiration hurried, jactitation, and struggles to leave the bed; skin cold and livid, delirium, with muttering of half finished expressions, subsultus tendinum and death at 3 o'clock, P. M.

In the management of this case, in the last few days of the patient's life, I had the advantage of the advice of Dr. I. Miller, of this place, and still later of Dr. Wm. Adair, of Cynthiana, Ky. No post mortem was made.

April 30th, 1853.

